# The prognostic significance of age in operated and non-operated colorectal cancer

**DOI:** 10.1186/s12885-015-1071-x

**Published:** 2015-02-25

**Authors:** Jing Li, Zhu Wang, Xin Yuan, Lichun Xu, Jiandong Tong

**Affiliations:** 1 Department of Oncology, The Second Clinical School of Yangzhou University (Yangzhou NO.1 People’s Hospital), Mid Hanjiang Road, Yangzhou, 225009 Jiangsu Province People’s Republic of China; 2 Research Center of Cancer Prevention and Treatment, Medical College of Yangzhou University, Number 11, Huaihai Road, Yangzhou, 225001 Jiangsu Province People’s Republic of China

**Keywords:** Colorectal cancer, Age, Survival analysis

## Abstract

**Background:**

The prognostic significance of age in colorectal cancer remains controversial. Our purpose was to determine the impact of age at diagnosis on cause- specific survival and overall survival in patients with colorectal cancer.

**Methods:**

Using Surveillance, Epidemiology, and End Results (SEER) population-based data, we identified 226,430 patients with colorectal cancer diagnosed between 1996 and 2005. Patients were separated into 10-year age groups. Five-year cancer cause-specific survival and overall survival data were obtained. Kaplan-Meier methods were adopted and multivariable Cox regression models were built for the analysis of long-term survival outcomes and risk factors.

**Results:**

In the operated group, those aged 51–60 had the best prognosis with 5-year cause-specific survival of 72.3% and 5-year overall survival of 68.3%.In the non-operated group, those of young age 15–30 had the best prognosis with 5-year cause-specific survival of 21.2% and 5-year overall survival of 18.2%, and there was continued worsening in cause-specific survival and overall survival with increasing age, except for a small increase in the 51–60 age group (P < 0.001). Multivariable analysis demonstrated a statistically significant disadvantage in cause-specific survival in patients older than 60 (P < 0.001), but the difference between the 51–60 age group and the younger age group (15–30, 31–40, 41–50) wasn’t statistically significant (P > 0.05) in both operated and non-operated patients.

**Conclusions:**

There was no apparent difference in survival in colorectal cancer patients 60 and younger, but in those older than 60 years, there was worsening in overall survival and cause-specific survival in both operated and non-operated patients.

**Electronic supplementary material:**

The online version of this article (doi:10.1186/s12885-015-1071-x) contains supplementary material, which is available to authorized users.

## Background

Colorectal cancer (CRC) is one of the most common malignancies and is ranked as the second leading cause of cancer-related deaths in the USA [1]. Median age at diagnosis is 69 years, and patients younger than 50 years represent approximately 10% of CRC [1-3]. The incidence of CRC has been increasing in younger patients over time [4]. While age plays a significant role in some cancers, such as thyroid, the notion that age is a significant prognostic factor in CRC has been controversial. For example, various studies have reported poorer prognosis among young patients with CRC [5-7], while other authors have demonstrated that young patients with CRC surgically treated appeared to have a higher cancer specific survival (CSS) rate than elderly ones [8-10]. Some studies showed more advanced stages in old patients [11] whereas others did not [12]. Furthermore, the current definition of young or elderly patients with CRC remains controversial.

Although the majority of studies in the literature used the cutoff age of 40 to denote young patients with CRC [5,9,13-15], some other studies have used cutoff age of 30 [15,16], 25 [17] or others [18-20]. The definition of an elderly patient has included cutoffs ages of 60 [21], 70 [22], 75 [23] and even 80 years [24,25]. The majority of studies was individually limited by surgical resection, but did not consider the prognostic significance of age on patients who were not surgically treated.

Our primary objective in this study was to determine the impact of age on the primary outcomes of CSS and overall survival (OS) among patients with CRC treated or not treated with surgical resection using data from SEER (Surveillance, Epidemiology, and End Results) database. Our secondary objectives was to determine whether there were differences in clinicopathological characteristics at the time of diagnosis for the various age groups.

## Methods

We used data from the SEER cancer registry to conduct this study. SEER, a population-based registry sponsored by the National Cancer Institute, collects information on cancer incidence and survival from 17 population-based cancer registries, including approximately 28% of the U.S. population [26]. SEER data contain no identifiers and are publicly available for studies on cancer-based epidemiology and health policy. The National Cancer Institute’s SEER*Stat software (Surveillance Research Program, National Cancer Institute SEER*Stat software, www.seer.cancer.gov/seerstat) (Version 8.1.2) was used to identify patients whose pathological diagnosis was invasive CRC (C18.0-20.9) between 1996 and 2005. Only patients of adult age (≥15 years) were included. Histology types were limited to adenocarcinoma (8150/3, 8210/3, 8261/3, 8263/3), mucinous adenocarcinoma (8480/3), and signet ring cell carcinoma (8490/3). Patients were excluded if they had in situ staging.

### Ethics statement

This study was in compliance with the Helsinki Declaration. An independent ethics committee/institutional review board at Yangzhou University approved our study. Data released from the SEER database do not require informed patient consent because they contain no identifiers and were publicly available. We have got permission to access the research data file in the SEER program by National Cancer Institute, USA and the reference number was 11756-Nov2013.

### Statistical analysis

Our use of the term “age” refers to “age at diagnosis” when not otherwise specified. Aside from ages 15–30 which were grouped together for a relatively small number of patients, other patients were stratified into 10-year age groups. Rather than dichotomizing patients as younger versus older, use of ten-year age groups allowed for a more detailed analysis of treatment by age. The primary endpoint of this study was CRC–cause-specific survival (CCSS) which was calculated from the date of diagnosis to the date of cancer-specific death and was shown as “SEER cause-specific survival” in SEER database. Overall survival (OS) was calculated from the date of diagnosis to the date of death, which was indicated as “Vital Status” in the SEER database. Age, sex, race, TNM stage, tumor location, tumor grade, histological type, CCSS and, OS were assessed. Adjuvant chemotherapy was not evaluated, as the SEER registry does not include this information. TNM classification was restaged according to the criteria described in the American Joint Committee on Cancer (AJCC) Cancer Staging Manual (7th edition, 2010).

Chi-square (*χ*2) tests were used for tests of independent parameters. Survival curves were generated using Kaplan-Meier estimates, and differences between curves were analyzed using the log-rank test. Multivariable Cox regression models were built for analysis of risk factors of survival outcomes. Exact 95% confidence intervals (CIs) for proportions were calculated. The nonlinear effect of age on the hazard ratio (HR) of CRC-specific mortality was assessed using quintic polynomial regression, with the R^2^ reported. All statistical analysis was done using the statistical software package SPSS for Windows, version 17 (SPSS Inc., Chicago, IL, USA). Statistical significance was set at two-sided P < 0.05.

## Results

### Clinicopathological differences between age groups

We identified 226,430 eligible patients with CRC in the SEER database during the 10-year study period (between 1996 and 2005). In the 15–30 age group, there were 1,181 patients; 5,333 in the 31–40 age group; 18,727 in the 41–50 age group; 39,125 in the 51–60 age group; 53,540 in the 61–70 age group; 64,642 in the 71–80 age group and 43,882 in the 80+ age group. The proportion of colon cancer patients and Caucasian patients gradually increased with age. Our 51–60, 61–70 and 71–80 age groups had a significantly larger proportion of grade I/II tumors at presentation (P < 0.001), as well as a significantly higher proportion of adenocarcinoma (P < 0.001), The proportions of patients receiving surgical resection was roughly same for the 15–30 to 71–80 age group with proportions varying from 90.2% to 91.4%, but it decreased to 84.1% for 80+ age group. The proportions of patients with stage I/II CRC gradually increased from 27.6% in the 15–30 age group to 46.0% in the 71–80 age group, but it decreased to 44.9% in the 80+ age group, which had highest proportion of unstaged patients (P < 0.05) (Table 1).

**Table 1:** **Characteristics of patients from SEER database by age**

	15-30	31-40	41-50	51-60	61-70	71-80	>80	P value
Characteristic	(n = 1181)	(n = 5333)	(n = 18727)	(n = 39125)	(n = 53540)	(n = 64642)	(n = 43882)	
Site								<0.001
Colon	878	3854	13619	29335	42067	52767	36839	
%	76.1%	73.6%	73.8%	76.1%	79.8%	83.0%	85.6%	
Rectum	276	1382	4823	9194	10625	10772	6205	
%	23.9%	26.4%	26.2%	23.9%	20.2%	17.0%	14.4%	
Sex								
Male	622	2881	10188	22876	30443	32157	16326	<0.001
%	52.7%	54.0%	54.4%	58.5%	56.9%	49.7%	37.2%	
Female	559	2452	8539	16249	23097	32485	27556	
%	47.3%	46.0%	45.6%	41.5%	43.1%	50.3%	62.8%	
Surgery Resection								<0.001
Yes	1073	4874	17025	35729	48909	58248	36890	
%	90.9%	91.4%	90.9%	91.3%	91.4%	90.2%	84.1%	
No	98	416	1594	3150	4318	6017	6647	
%	8.3%	7.8%	8.5%	8.1%	8.1%	9.3%	15.2%	
Unknown	10	43	105	243	292	347	316	
%	0.8%	0.8%	0.6%	0.6%	0.5%	0.5%	0.7%	
Grade								<0.001
I/II	687	3555	13196	28331	39003	46869	30960	
%	58.2%	66.7%	70.5%	72.4%	72.8%	72.5%	70.6%	
III/IV	358	1232	3589	6649	9212	11727	8526	
%	30.3%	23.1%	19.2%	17.0%	17.2%	18.1%	19.4%	
Unknown	136	546	1942	4145	5325	6046	4396	
%	11.5%	10.2%	10.4%	10.6%	9.9%	9.4%	10.0%	
Race								<0.001
Caucasian	865	3943	14062	30358	42785	54227	38195	
%	73.2%	73.9%	75.1%	77.6%	79.9%	83.9%	87.0%	
African American	158	726	2689	5190	6144	5550	3101	
%	13.4%	13.6%	14.4%	13.3%	11.5%	8.6%	7.1%	
Others*	153	637	1864	3361	4358	4604	2458	
%	13.0%	11.9%	10.0%	8.6%	8.1%	7.1%	5.6%	
Unknown	5	27	112	216	253	261	128	
%	0.4%	0.5%	0.6%	0.6%	0.5%	0.4%	0.3%	
Histological Type								<0.001
Adenocarcinoma	833	4363	16225	34740	47536	56905	38431	
%	72.2%	82.5%	86.9%	89.0%	88.9%	88.1%	87.7%	
Mucinous/Signet-ring cancer	320	927	2440	4308	5927	7655	5384	
%	27.8%	17.5%	13.1%	11.0%	11.1%	11.9%	12.3%	
AJCC stage								<0.001
I- II	326	1857	6736	15467	23031	29743	19712	
%	27.6%	34.8%	36.0%	39.5%	43.0%	46.0%	44.9%	
III -IV	705	2828	9432	17834	22753	25328	15786	
%	59.7%	53.0%	50.4%	45.6%	42.5%	39.2%	36.0%	
Unknown	150	648	2559	5824	7756	9571	8384	
%	12.7%	12.2%	13.7%	14.9%	14.5%	14.8%	19.1%	

*Including American Indian/AK Native, Asian/Pacific Islander.

### Impact of age on survival outcomes in patients with CRC

We observed two significant findings. First, in the operated group, those aged 51–60 had the best prognosis with a 5-year CCSS of 72.3% and a 5-year OS of 68.3%. Patients in the 15–30 age group had reduced CCSS and OS rates, but they were better than those in the 80+ age group, especially for OS. Second, in the non-operated group, there was continued worsening in CCSS and OS with increasing age, except for a slight increase in the 51–60 age group. The 5-year CCSS and OS rates decreased from 21.2% to 11.9% and from 18.2% to 4.3% in the 15–30 compared with 80+ age group, respectively (Table 2).

**Table 2:** **Long time survival rate (CSS/OS) in colorectal cancer by age**

	15-30	31-40	41-50	51-60	61-70	71-80	>80	P value
**Total**	(n = 1181)	(n = 5333)	(n = 18727)	(n = 39125)	(n = 53540)	(n = 64642)	(n = 43882)	
CSS								<0.001
1-year CCS	84.6%	88.8%	88.8%	88.7%	86.8%	82.8%	74.2%	
3-year CCS	66.4%	72.7%	73.3%	75.1%	74.1%	70.4%	60.0%	
5-year CCS	58.5%	65.7%	66.3%	68.0%	67.6%	64.0%	53.5%	
OS								
1-year OS	83.5%	87.9%	87.8%	87.2%	83.9%	77.3%	63.8%	
3-year OS	64.8%	71.0%	71.6%	72.4%	68.9%	60.5%	43.0%	
5-year OS	56.6%	63.6%	63.5%	64.0%	60.0%	49.9%	30.5%	
**Operated group**	(n = 1073)	(n = 4874)	(n = 17025)	(n = 35729)	(n = 48909)	(n = 58248)	(n = 36890)	
5-year CCS								<0.001
1-year CCS	88.4%	91.7%	92.3%	92.1%	90.6%	87.3%	80.6%	
3-year CCS	70.4%	76.5%	77.9%	79.5%	78.4%	75.2%	67.0%	
5-year CCS	62.1%	69.6%	70.7%	72.3%	71.8%	68.6%	60.1%	
OS								
1-year OS	87.4%	90.9%	91.6%	90.8%	88.0%	82.3%	70.7%	
3-year OS	69.1%	75.1%	76.3%	76.8%	73.4%	65.5%	49.5%	
5-year OS	60.4%	67.6%	68.3%	68.3%	64.2%	54.2%	35.3%	
**Non-operated group**	(n = 98)	(n = 416)	(n = 1594)	(n = 3150)	(n = 4318)	(n = 6017)	(n = 6647)	
CSS								
1-year CCS	42.7%	55.6%	50.2%	50.3%	43.0%	37.4%	36.5%	
3-year CCS	25.2%	27.2%	23.4%	25.6%	23.3%	20.7%	17.0%	
5-year CCS	21.2%	20.3%	18.4%	19.7%	18.3%	16.7%	11.9%	
OS								
1-year OS	41.1%	54.0%	48.1%	47.6%	38.7%	30.7%	26.3%	
3-year OS	21.6%	25.0%	21.8%	23.0%	19.2%	14.2%	8.4%	
5-year OS	18.2%	18.0%	16.4%	17.0%	14.0%	9.8%	4.3%	

**Figure 1:**
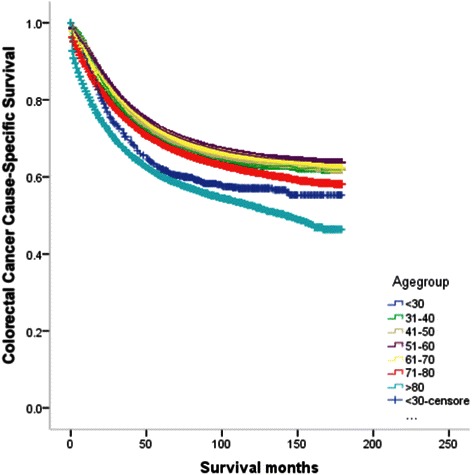
**Survival curves according to each age subgroups in colorectal patients in operated group.**

**Figure 2:**
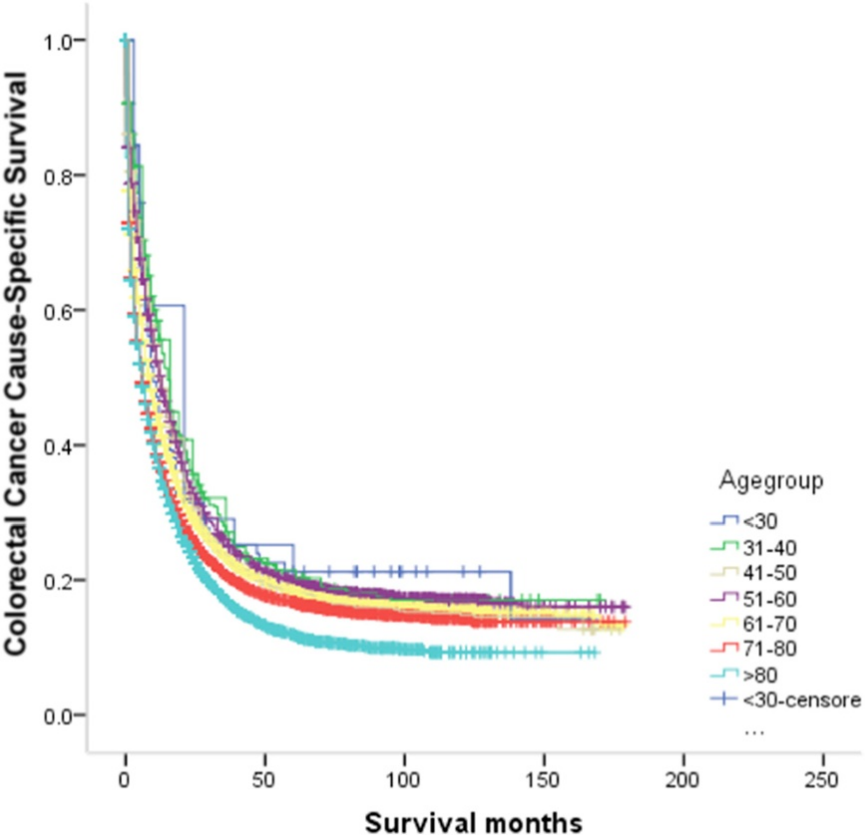
**Survival curves according to each age subgroups in colorectal patients in non-operated group.**

**Figure 3:**
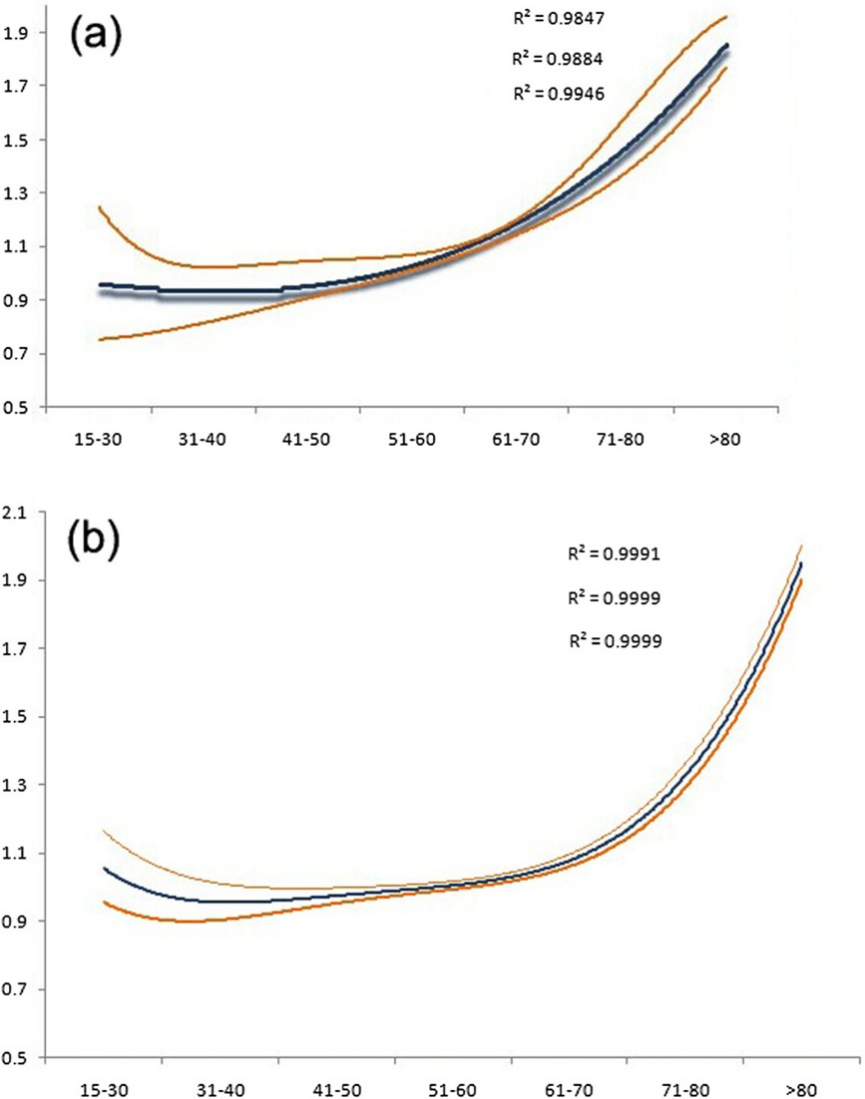
**Estimates of hazard ratios (HRs) of colorectal cancer-specific mortality changing with age for (a) operated and (b) non-operated group using quintic polynomial regression.** The solid blue lines represent the estimates of HRs, whereas the dotted orange lines represent the 95% confidence intervals. All R2 values are reported.

The results of the univariate survival analysis and multivariable Cox proportional hazard regression analysis of age and various covariates with respect to CCSS in operated and non-operated groups are shown in Tables 3, 4, 5 and 6, and Figures 1 and 2. In the operated group, multivariable analysis demonstrated statistically significant increases in HR after age 60. Patients in the 61–70 age group were 1.1 times more likely to die of cancer than patients in the 51–60 age group. The risk was significantly higher for the patients in the 71–80 age group who were 1.3 times more likely to die of cancer than those in the 51–60 age group. Finally, the risk was higher still for patients in the 80+ age group who were 1.9 times more likely to die of cancer than 51–60 year old patients. Patients in the elderly age groups (61–70, 71–80, and 80+) had significantly worse survival than those in the 51–60 age group (P < 0.001), but the difference in survival between those in the 51–60 age group compared with the other three younger age groups (15-30, 31-40, 41-50) wasn’t statistically significant (P > 0.05) (Table 5). The plot of HRs in this subgroup showed a hook-shaped curve and HRs sharply increased in the above 60 age group (Table 5, Figure 3a).

**Table 3:** **Univariate survival analyses of CRC patients in operated group according to various clinicopathological variables**

Variable	n	5-year CCSS (%)	Log rank*χ*^2^test	P
Years of diagnosis			65.374	<0.001
1996-2000	75517	67.5%		
2001-2005	127231	69.5%		
Sex			2.520	0.112
Male	103083	69.0%		
Female	99665	68.5%		
Site			122.381	<0.001
Colon	127618	68.2%		
Rectum	35530	71.6%		
Age			1112.554	<0.001
15-30	1073	62.1%		
31-40	4874	69.6%		
41-50	17025	70.7%		
51-60	35729	72.3%		
61-70	48909	71.8%		
71-80	58248	68.6%		
>80	36890	60.1%		
Race			0.036	0.850
Caucasian	166019	69.2%		
African American	20149	61.6%		
Others*	16580	72.4%		
Pathological grading			812.382	<0.001
I- II	150521	72.0%		
III-IV	38018	52.7%		
Unknown	14209	77.2%		
Histological Type			1162.165	<0.001
Adenocarcinoma	177748	70.0%		
Mucinous/Signet ring cancer	25000	59.7%		
AJCC stage			16776.287	<0.001
I	37364	92.6%		
II	59213	81.5%		
III	54234	61.3%		
IV	27707	12.6%		
Unknown	24230	81.8%		
No. of LNs dissected			337.494	<0.001
<12	116783	69.6%		
≥12	80248	69.0%		
Unknown	5717	47.7%		

*Including other (American Indian/AK Native, Asian/Pacific Islander) and unknowns.

**Table 4:** **Univariate survival analyses of CRC patients in non-operated group according to various clinicopathological variables**

Variable	n	5-year CCSS (%)	Log rank*χ*^2^test	P
Years of diagnosis			8.182	0.004
1996-2000	7617	16.6%		
2001-2005	14623	16.5%		
Sex			19.224	<0.001
Male	11622	16.7%		
Female	10618	16.3%		
Site			443.334	<0.001
Colon	15119	14.8%		
Rectum	7121	20.1%		
Age			257.702	<0.001
15-30	98	21.2%		
31-40	416	20.3%		
41-50	1594	18.4%		
51-60	3150	19.7%		
61-70	4318	18.3%		
71-80	6017	16.7%		
>80	6647	11.9%		
Race			26.525	<0.001
Caucasian	17250	16.5%		
African American	3218	12.2%		
Others*	1772	23.9%		
Pathological grading			812.382	<0.001
I- II	11275	19.9%		
III-IV	3113	9.2%		
Unknown	7852	14.4%		
Histological Type			116.240	<0.001
Adenocarcinoma	20362	17.2%		
Mucinous/Signet ring cancer	1878	9.3%		
AJCC stage			1115.463	<0.001
I	82	74.8%		
II	189	64.7%		
III	128	46.0%		
IV	12202	3.5%		
Unknown	9639	32.8%		

*Including other (American Indian/AK Native, Asian/Pacific Islander) and unknowns.

**Table 5:** **Multivariate Cox model analyses of prognostic factors of CRC in operated group**

Variable	Hazard ratio	95%CI	P
Years of diagnosis			<0.001
1996-2000	1.000	Reference	
2001-2005	0.944	0.930-0.959	
Site			<0.001
Colon	1.000	Reference	
Rectum	1.044	1.023-1.066	
Age			<0.001
15-30	1.055	0.955-1.165	0.294
31-40	0.954	0.906-1.005	0.077
41-50	0.979	0.948-1.011	0.203
51-60	1.000	Reference	
61-70	1.082	1.056-1.109	<0.001
71-80	1.325	1.294-1.356	<0.001
>80	1.951	1.902-2.002	<0.001
Pathological grading			<0.001
I- II	1.000	Reference	
III-IV	1.443	1.418-1.468	<0.001
Unknown	0.913	0.880-0.948	<0.001
Histological Type			<0.001
Adenocarcinoma	1.000	Reference	
Mucinous/Signet ring cancer	1.200	1.175-1.226	
AJCC stage			<0.001
I- II	1.000	Reference	
III- IV	4.880	4.791-4.971	<0.001
Unknown	1.170	1.130-1.210	<0.001
No. of LNs dissected			<0.001
<12	1.000	Reference	
≥12	0.793	0.780-0.806	<0.001
Unknown	2.269	2.183-2.358	<0.001

**Table 6:** **Multivariate Cox model analyses of prognostic factors of CRC in non-operated group**

Variable	Hazard ratio	95%CI	P
Years of diagnosis			<0.001
1996-2000	1.000	Reference	
2001-2005	0.869	0.840-0.899	
Sex			0.881
Male	1.000	Reference	
Female	0.997	0.965-1.031	
Site			<0.001
Colon	1.000	Reference	
Rectum	0.842	0.814-0.872	
Age			<0.001
15-30	0.975	0.761-1.251	0.845
31-40	0.886	0.784-1.002	0.055
41-50	1.021	0.951-1.097	0.567
51-60	1.000	Reference	
61-70	1.185	1.121-1.253	<0.001
71-80	1.463	1.388-1.543	<0.001
>80	1.859	1.762-1.961	<0.001
Race			<0.001
Caucasian	1.000	Reference	
African American	1.160	1.109-1.214	<0.001
Others*	0.871	0.818-0.927	<0.001
Pathological grading			<0.001
I- II	1.000	Reference	
III-IV	1.430	1.365-1.498	<0.001
Unknown	1.141	1.100-1.184	<0.001
Histological Type			0.001
Adenocarcinoma	1.000	Reference	
Mucinous/Signet ring cancer	1.112	1.050-1.177	
AJCC stage			<0.001
I- II	1.000	Reference	
III- IV	7.808	6.260-9.739	<0.001
Unknown	2.735	2.192-3.412	<0.001

*Including other (American Indian/AK Native, Asian/Pacific Islander) and unknowns.

In the non-operated groups, multivariable analysis demonstrated similar results with the operated group. The risk of death from CRC continued to increase in the above 60 age group, such that patients in the 61–70, 71–80 and 80+ age groups were 1.2, 1.5 and 1.9 times, respectively more likely to die of cancer than patients in the 51–60 age group. Those in the 15-30, 31-40, 41-50 age groups experienced similar CCSS compared with those in the 51–60 age group (P > 0.05) (Table 6). The curves of the HRs in these groups was steadily constant until the age of 40, when the HR started to apparently increase with increasing age (Table 6 and Figure 3b).

## Discussion

Conflicting results have been reported regarding whether age affects the prognosis of CRC. In general, it is assumed that young patients have a higher prevalence of mucinous or poorly differentiated tumors including signet ring carcinoma and later stage and elderly patients have a higher percentage of comorbidities and emergency surgeries, all of which means poorer prognosis compared with others. Whether age itself is an independent survival factor is unknown. Previous studies have demonstrated that age influences CSS in certain cancer types. One example is well differentiated thyroid cancer for which the American Joint Committee on Cancer (AJCC) incorporates age into its staging criteria: patients less than 45 years of age with well differentiated thyroid cancer cannot be diagnosed with stage III or IV disease [27].

In our study cohort, we found that patients in the 51–60 age group had a relatively good prognosis, and the difference with elderly age groups (61-70-, 71–80, 80+) was statistically significant, but the difference between the 51–60 and younger groups was not significant. This result was validated in both operated and non-operated age groups. Although young patients always had a higher prevalence of later stage (stage III/IV) and mucinous, signet-ring and poorly differentiated tumors which tended to have a poorer prognosis compared with well and moderately differentiated tumors [28], the surgical resection rate and survival rate were comparable with other age groups in our study. What is interesting is that if patients didn’t undergo surgical resection, the younger they were, the better was the prognosis.

Generally, CRC is thought to be a malignancy affecting mostly the elderly, with more than 90% of patients being diagnosed after age 55, and our study indicated that it seems reasonable to use 60 years as the cutoff between young and elderly patients. We then conducted an analysis comparing clinicopathological and survival analysis comparing groups of patients above and below 60 years of age, Young patients with CRC aged 60 and below had unfavorable clinicopathological characteristics, but they still had a higher CSS (*χ*2 = 631.268, P < 0.001). (Additional file 1: Table S1 and Additional file 2: Figure S1).

In general, young patients always had a good performance status, which is essential for the success of chemotherapy [29] and extensive lymphadenectomy. Clinicians are more inclined to use all therapeutic options, such as combination chemotherapy and surgery in young patients than elderly ones because they are in better health and are more likely to tolerate toxicities associated with chemotherapy [10,30,31]. In a retrospective large multi-institutional study, chemotherapy use in patients with stage III disease decreased with increasing age, with patients >80 years receiving adjuvant chemotherapy in only 25.6% of cases in comparison with 82.4% of cases in patients <40. For select patients with stage II disease, younger patients more frequently received chemotherapy than older patients (69.2% for those <40 year, 46.0% for those 40–50, 27.0% for those 50–80 and 5.6% for those >80 years of age), and similar results were found in rectal cancer [32].

Increased adjuvant therapy use in younger patients may partially account for stage-specific increases in survival. Moreover, the sharp decline in the use of adjuvant therapy with increasing age could not be justified by the effect of comorbidities, treatment toxicity, short natural life expectancies, and health care [32,33]. In several institutional series, young patients more often received regional or surgical therapy compared with older patients [13,34,35]. Conversely, poor tolerance to treatment due to poor performance status or the presence of other comorbid medical conditions may contribute to inferior survival of older patients [12]. *Chagpar* et al. found that, among patients with stage III disease, older age was associated with under treatment, independent of preexisting comorbidities, as well as other clinical pathologic and socioeconomic factors [36].

When concerning metastatic CRC, several studies have shown a significant improvement in survival when comparing patients managed with primary tumor resection to those managed with chemotherapy alone [31,37-39], but in fact elderly patients are less likely to receive palliative surgery. Our data demonstrated that patients in the 80+ age group had an extremely low surgical resection rate although they had relatively good clinicopathological characteristics. Less aggressive treatment offered to patients with limited comorbidities was likely to impact on their outcome [40].

Young patients also have a higher proportion of tumors demonstrating microsatellite instability, which are associated with a better prognosis [41]. Although survival of patients with advanced CRC has significantly increased in clinical trials incorporating new therapeutic agents [42], a meta-analysis comparing younger and older patients with advanced CRC enrolled in randomized clinical trials of newer chemotherapy agents between 1995 and 2004 demonstrated equal survival in both groups [43]. In fact, patients enrolled in clinical trials are always strictly selected and under thorough supervision. Our analysis of the SEER data demonstrated that the younger the patients were, the better their survival, even for patients who received no surgical therapy.

This study adds to current knowledge by answering more in-depth research questions about age and prognosis through analysis large population-based data from the SEER database, however, it had several potential limitations. First, the SEER database only had limited information on tumor factors, which could affect survival analysis. Second, concerning treatment modalities, the SEER definition of surgery does not separate treatment with palliative intent from that with curative intent. Thus, the beneficial effect of surgery on survival may be underestimated, specifically for those patients who underwent radical resection. Finally, the SEER database does not include information on comorbidities which limits our ability to calculate the impact of comorbid conditions on CSS. Various studies on CRC surgery and treatment outcomes show a progressive increase in post-operative morbidity and mortality with advancing age [44,45], which are likely to impact their short-term survival. Moreover, some studies have shown that postoperative morbidity had a negative impact on long-term outcomes following radical surgery of various tumors [46-48]. Still, our study has sufficient power for a larger population-based study.

## Conclusions

In conclusion, our study demonstrated that in the operated group, those aged 51–60 had the best prognosis, while in the non-operated groups, those of very young age 15–30 had the best prognosis. Both CCS and OS continued to decline with further advances in age after 60 as exemplified by worsening survival in the >60 age group. Recognition of these findings is important for clinicians who must consider if age should be incorporated into their assessments and treatment decisions for patients with CRC.

## Additional files

Additional file 1: Table S1. Characteristics of Patients from SEER Database by age. **Table S2.** Multivariate Cox model analyses of prognostic factors of CRC.
Additional file 2: Figure S1. Survival analysis comparing groups of patients with colorectal cancer above and below 60 years of age.

## Abbreviations

CRC: Colorectal cancer
SEER: National Cancer Institute’s Surveillance, Epidemiology, and End Results
AJCC: American Joint Committee on Cancer
CSS: Cause-specific survival rate
OS: Overall survival
