# Supplementary material for: The prognostic significance of age in operated and non-operated colorectal cancer
Source: BMC Cancer. 2015 Feb 25;15:83. doi: 10.1186/s12885-015-1071-x (PMC4345025; doi:10.1186/s12885-015-1071-x)
Supplement: Additional file 1: Table S1. — Characteristics of Patients from SEER Database by age. Table S2. Multivariate Cox model analyses of prognostic factors of CRC. [file 12885_2015_1071_MOESM1_ESM.doc]

**Table S1. Characteristics of Patients from SEER Database by age**

|  | Total | Young Group | Elderly Group | | P value |
| --- | --- | --- | --- | --- | --- |
|  |  | (<60) | (≥60) | |  |
| Characteristic | (n=226430) | (n=64366) | (n=162064) | |  |
| Site |  |  |  | P<0.001 | |
| Colon | 183153 | 48691 | 134462 |  | |
| Rectum | 43277 | 15675 | 27602 |  | |
| Sex |  |  |  | P<0.001 | |
| male | 115493 | 36567 | 78926 |  | |
| female | 110937 | 27799 | 83138 |  | |
| Surgery Resection |  |  |  | P<0.001 | |
| Yes | 202748 | 58701 | 144047 |  | |
| No | 22240 | 5258 | 16982 |  | |
| Unknown | 1356 | 401 | 955 |  | |
| Race |  |  |  | P<0.001 | |
| Caucasian | 184435 | 49228 | 135207 |  | |
| African American | 23558 | 8763 | 14795 |  | |
| Others | 18437 | 6375 | 12062 |  | |
| [Pathological grading](http://dict.cn/pathological grading) |  |  |  | P<0.001 | |
| I/II | 162601 | 45769 | 116832 |  | |
| III/IV | 41293 | 11828 | 29465 |  | |
| Unknown | 22536 | 6769 | 15767 |  | |
| Histological Type |  |  |  | P<0.001 | |
| Adenocarcinoma | 199469 | 56371 | 143098 |  | |
| Mucinous/  Signet-ring cancer | 26961 | 7995 | 18966 |  | |
| AJCC stage |  |  |  | P<0.001 | |
| I- II | 96872 | 24386 | 72486 |  | |
| III -IV | 94666 | 30799 | 63867 |  | |
| Unknown | 34892 | 9181 | 25711 |  | |

**TABLE S2.** Multivariate Cox model analyses of prognostic factors of CRC

| **Variable** | **Hazard Ratio** | **95%CI** | **P** |
| --- | --- | --- | --- |
| Sex |  |  | 0.115 |
| male | 1.000 | Reference |  |
| female | 0.989 | 0.976-1.003 |  |
| Site |  |  | <0.001 |
| Colon | 1.000 | Reference |  |
| Rectum | 0.952 | 0.936-0.969 |  |
| Surgery Resection |  |  | <0.001 |
| Yes | 1.000 | Reference |  |
| No | 5.008 | 4.909-5.108 |  |
| Unknown | 2.918 | 2.710-3.141 |  |
| Race |  |  | <0.001 |
| Caucasian | 1.000 | Reference |  |
| African American | 1.230 | 1.205-1.255 |  |
| Others* | 0.839 | 0.817-0.861 |  |
| Age |  |  | <0.001 |
| <60 | 1.000 | Reference |  |
| ≥60 | 1.408 | 1.387-1.430 |  |
| [Pathological grading](http://dict.cn/pathological grading) |  |  | <0.001 |
| I- II | 1.000 | Reference |  |
| III-IV | 1.463 | 1.439-1.487 |  |
| Unknown | 1.094 | 1.069-1.119 |  |
| Histological Type |  |  | <0.001 |
| Adenocarcinoma | 1.000 | Reference |  |
| Mucinous/Signet ring cancer | 1.187 | 1.164-1.210 |  |
| AJCC stage |  |  | <0.001 |
| I- II | 1.000 | Reference |  |
| III- IV | 4.611 | 4.528-4.695 |  |
| Unknown | 1.418 | 1.381-1.456 |  |
| *including other (American Indian/AK Native, Asian/Pacific Islander) and unknowns. | | | |
