# Supplementary figures and images for: The prognostic significance of age in operated and non-operated colorectal cancer
Source: BMC Cancer. 2015 Feb 25;15:83. doi: 10.1186/s12885-015-1071-x (PMC4345025; doi:10.1186/s12885-015-1071-x)

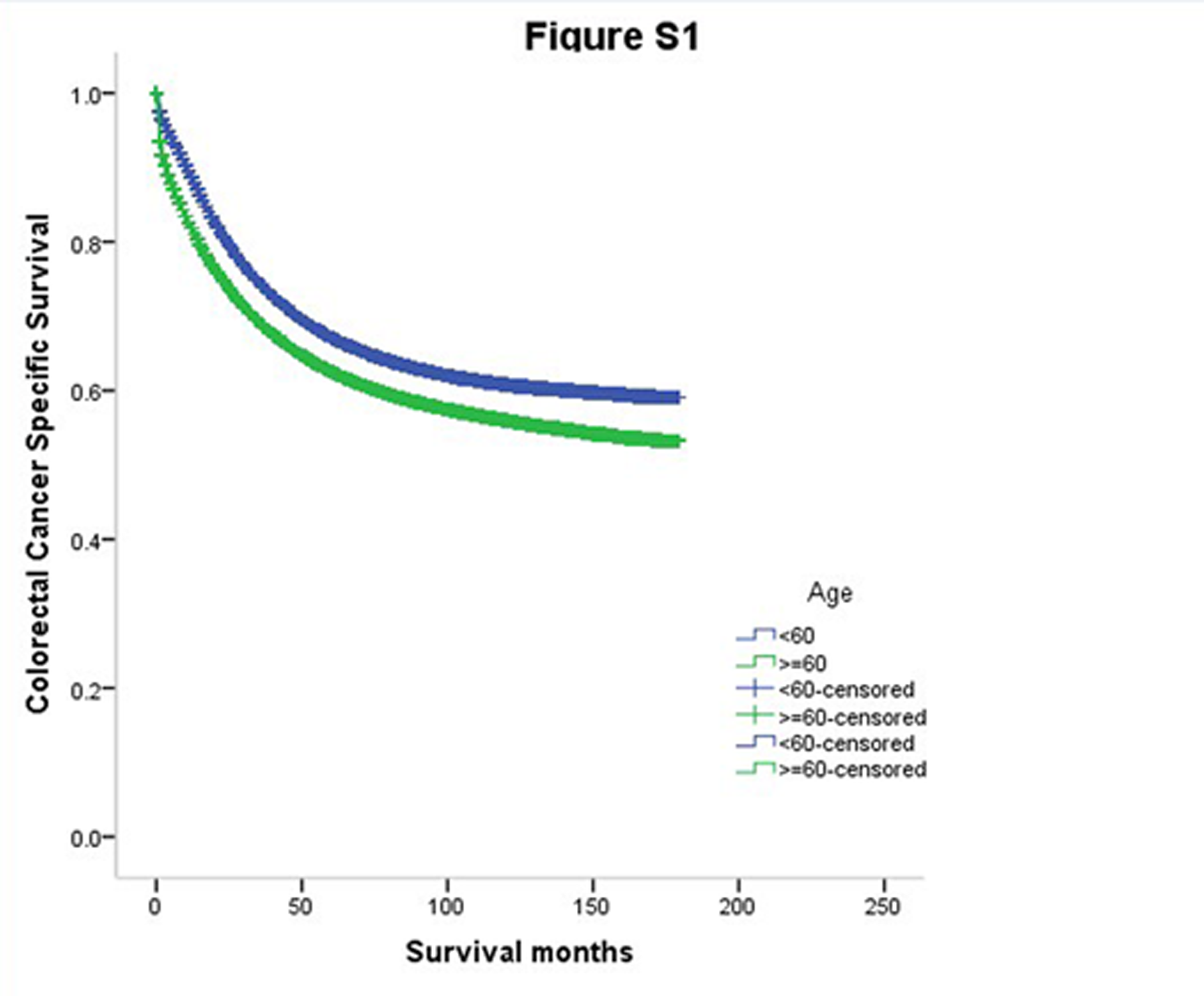

Supplement: Additional file 2: Figure S1. — Survival analysis comparing groups of patients with colorectal cancer above and below 60 years of age. [file 12885_2015_1071_MOESM2_ESM.tiff]
